# Mass distribution of free insecticide-treated nets do not interfere with continuous net distribution in Tanzania

**DOI:** 10.1186/1475-2875-13-196

**Published:** 2014-05-27

**Authors:** Ikenna C Eze, Karen Kramer, Amina Msengwa, Renata Mandike, Christian Lengeler

**Affiliations:** 1 Swiss Tropical and Public Health Institute, P.O. Box, 4002, Basel, Switzerland; 2 University of Basel, Basel, Switzerland; 3 National Malaria Control Programme, Dar es Salaam, Tanzania; 4 University of Dar es Salaam, Dar es Salaam, Tanzania

**Keywords:** Malaria, Malaria control, Voucher scheme, Long-lasting insecticidal nets, Ecological study, Tanzania

## Abstract

**Background:**

To protect the most vulnerable groups from malaria (pregnant women and infants) the Tanzanian Government introduced a subsidy (voucher) scheme in 2004, on the basis of a public-private partnership. These vouchers are provided to pregnant women at their first antenatal care visit and mothers of infants at first vaccination. The vouchers are redeemed at registered retailers for a long-lasting insecticidal net against the payment of a modest top-up price. The present work analysed a large body of data from the Tanzanian National Voucher Scheme, focusing on interactions with concurrent mass distribution campaigns of free nets.

**Methods:**

In an ecologic study involving all regions of Tanzania, voucher redemption data for the period 2007 2011, as well as data on potential determinants of voucher redemption were analysed. The four outcome variables were: pregnant woman and infant voucher redemption rates, use of treated bed nets by all household members and by under- five children. Each of the outcomes was regressed with selected determinants, using a generalized estimating equation model and accounting for regional data clustering.

**Results:**

There was a consistent improvement in voucher redemption rates over the selected time period, with rates >80% in 2011. The major determinants of redemption rates were the top-up price paid by the voucher beneficiary, the retailer- clinic ratio, and socio-economic status. Improved redemption rates after 2009 were most likely due to reduced top-up prices (following a change in policy). Redemption rates were not affected by two major free net distribution campaigns. During this period, there was a consistent improvement in net use across all the regions, with rates of up to 75% in 2011.

**Conclusion:**

The key components of the National Treated Nets Programme (NATNETS) seem to work harmoniously, leading to a high level of net use in the entire population. This calls for the continuation of this effort in Tanzania and for emulation by other countries with endemic malaria.

## Background

**Figure 1:**
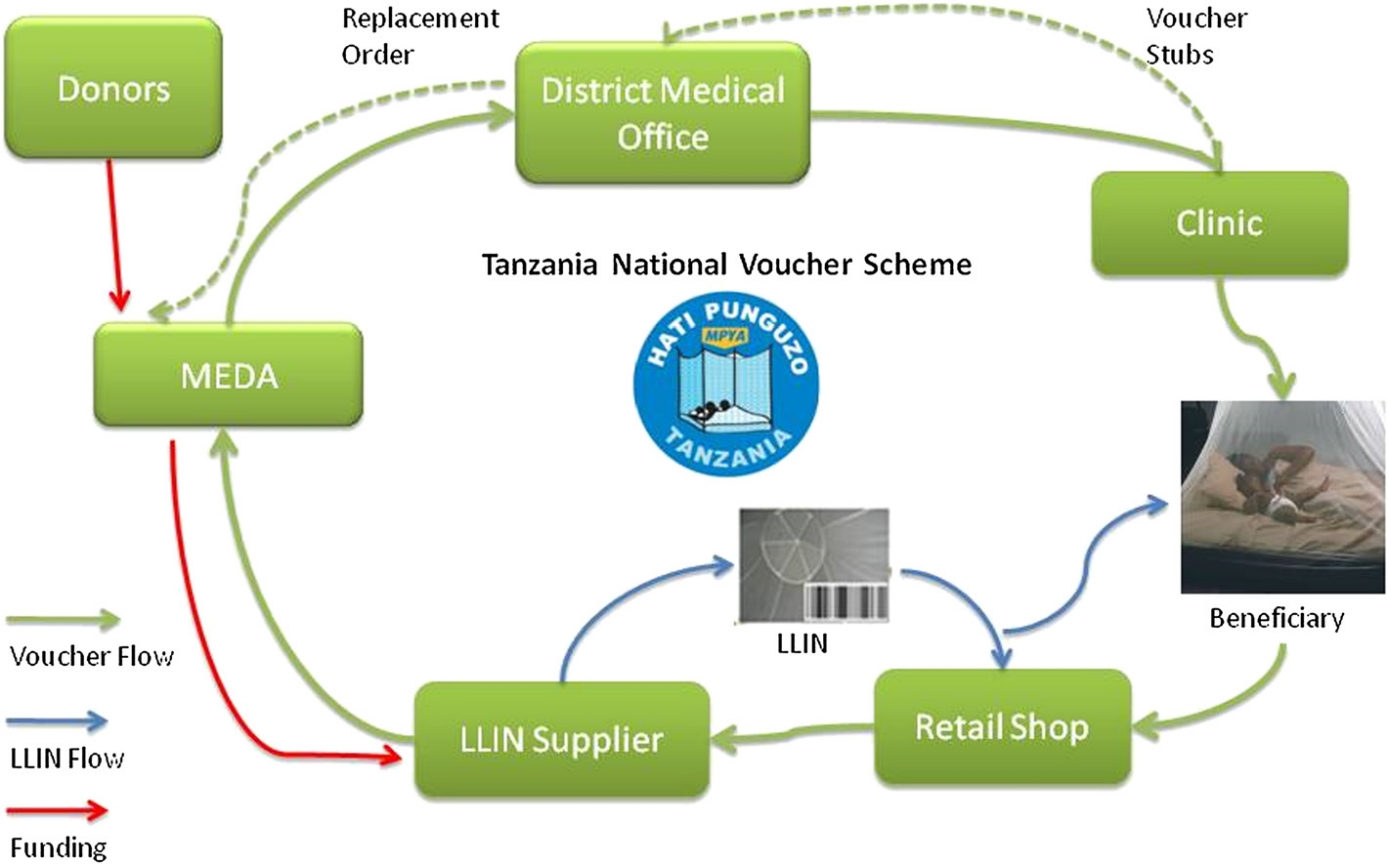
**TNVS Voucher/LLIN cycle.** Arrows represent direction of voucher and net flow. LLIN: Long-Lasting Insecticidal Nets; MEDA: Mennonite Economic Development Associates.

Malaria continues to be a large burden of disease in the world [1]. In Tanzania alone it kills about 60,000 children annually [2], with enormous economic implications [3]. The burden of malaria is most felt across the most vulnerable groups, which include pregnant women and under-five children. In the light of this, the Tanzanian Government developed a subsidy (voucher) scheme to distribute long-lasting insecticide-treated nets (LLIN) to pregnant women and under-five children in a public-private partnership called the Tanzanian National Voucher Scheme (TNVS) [4,5]. This scheme, which has a very active private sector involvement, started in 2004 following a number of projects aimed at making ITNs more affordable for the population. SMARTNET was the biggest of these projects and contributed to a strong development of the private sector for nets from 2002 until 2007 [4]. The Infant Vouchers (IV) are given to mothers/fathers of infant coming for their first vaccination in Mother-and-Child clinics, while the Pregnant Woman Vouchers (PWV) are given to pregnant women in antenatal clinics. The women/parents/ guardians later redeem the vouchers for an LLIN from accredited retailers with a small top-up payment (Figure 1). The TNVS is part of the National Insecticide-Treated Nets Programme (NATNETS), which involves also other net distribution strategies such as the mass campaigns of free LLINs [6,7].

The vouchers are handled by a non-governmental contractor (Mennonite Economic Development Associates - MEDA), which also track the vouchers across the entire cycle. The vouchers are dispatched from Dar es Salaam to the District Medical Offices (DMOs) on request, and are labeled “dispatched vouchers”. The DMOs send the vouchers to the clinics on request, as the “sent out vouchers”. The clinics give the vouchers to pregnant women and mothers of infants, which are then tagged as “issued vouchers”. The women use these issued vouchers to redeem an LLIN at a retail shop upon payment of a top-up fee, which varied over time and was set to TZS 500 (about US$ 0.40) in 2009. These vouchers are “exchanged vouchers”. The retailers submit the redeemed vouchers to the manufacturers (suppliers) in exchange for more LLINs as “swapped vouchers”. The manufacturer then returns them to MEDA for payment, and at this point they are “returned vouchers”. The returned vouchers are then matched with the corresponding voucher stubs that have been returned from the clinics for final reconciliation. The matched vouchers become the “redeemed vouchers” and it is on this basis that the manufacturers are paid (Figure 1), while fraudulent vouchers are voided. The TNVS started with the PWV in 2004 and included the IV in 2006. By December 2013, over four million infant vouchers and nine million pregnant woman vouchers had been redeemed for a LLIN [5].

Since 2013, an e-voucher scheme involving the use of mobile phones to transmit codes used in the voucher supply chain is being gradually introduced into the TNVS to replace the paper voucher system. The e-voucher became necessary for three reasons: (i) to enhance the timely voucher usage by the beneficiaries, (ii) to help reduce the financial liability created by unredeemed vouchers, (iii) to allow real-time data handling and eliminating voucher stock-outs at clinics. It is also useful in reducing LLIN stock-outs at retailers and in better controlling voucher misuse and fraud.

The TNVS undergoes continuous monitoring and evaluation in the forms of household surveys [8-10], retail audits [11,12], voucher tracking [13,14] and qualitative studies [15,16]. A 2003 evaluation of the precursor of TNVS in the frame of the KINET project in two rural districts of southern Tanzania showed high voucher return rates but low awareness and voucher use two years after initiation of the programme. This prompted more activities in Behaviour Change Communication (BCC) [17]. Mulligan and colleagues [18] explored the cost effectiveness of the scheme, whereas Tami *et al*. explored issues surrounding voucher misuse [19]. In 2007, Marchant and colleagues assessed coverage equity of the voucher scheme and proposed steps to improve deficiencies [20], whereas Donaldson and Thiede, evaluated in 2011 the general performance of the voucher scheme, without investigating the determinants of voucher redemption across the regions of Tanzania [5].

Following voucher redemption trends and assessing determinants of redemption rates is crucial in ensuring effectiveness of the TNVs and the efficient use of resources. In addition, there was considerable concern as to whether mass distributions of free LLINs, which took place between 2009 and 2011, would negatively affect the demand for vouchers and their redemption, possibly leading to a loss of interest in the TNVS by the private net sector, which is crucial for its functioning. This was studied, in order to support the future planning of NATNETS components.

## Methods

This was an ecologic study involving a time series analysis of the key factors influencing voucher redemption rates and bed net coverage in Tanzania between 2005 and 2011. The unit of data collection was the administrative region (the second administrative level in the country). As of 2011, mainland Tanzania had 21 regions and 120 districts. District-level analysis was not possible because of lack of uniformity in the available data.

### Study outcomes

The primary outcomes were PWV and IV redemption rates, whereas the secondary outcomes were household (all ages) and under-five use of treated bed nets. Voucher redemption rates were expressed as percentages and were defined as total number of vouchers redeemed divided by the total number of stubs returned in a given time period. Bed net use was defined as sleeping under an insecticide-treated bed net the night preceding the household surveys, and the rate of bed net use was expressed as a percentage of all relevant population in the relevant household survey. “Household bed net use” considered everyone in the household regardless of age and sex, while under-five bed net use only considered children below the age of five years. The study outcomes, data sources and years of availability are shown in Table 1. Table 2 summarizes the potential determinants, data source, years of data availability and their hypothesized effect on the key outcomes.

**Table 1:** Outcomes of present study

**Outcome**	**Years available**	**Source**
Infant Voucher redemption rates	2005-2011	MEDA
Pregnant Woman Voucher redemption rates	2007-2011	MEDA
Household bed net use	2005-2010	NMCP
Under-five bed net use	2005-2010	NMCP

MEDA: Mennonite Economic Development Associates; NMCP: National Malaria Control Programme.

**Table 2:** Selected determinants of voucher redemption rates and bed net use, with data sources

**Variable**	**Years available**	**Hypothesized effect on outcome**	**Source**
Retailer-clinic Ratio	2005-2011	Increase in the ratio will increase redemption rates	MEDA
Average top-up price	2005-2011	Increase in top-up price will decrease redemption rates	NMCP
Under-5 coverage campaign (U5CC)	2009-2010	Free net campaign will decrease redemption rates	MEDA
Universal coverage Campaign (UCC)	2010-2011	Free net campaign will decrease redemption rates	MEDA
Prevalence of malaria	2007, 2012	Higher prevalence will increase redemption rates	THMIS
Exposure to mass media	2010	Higher exposure to media will increase redemption rates	TDHS
Relative wealth index (of region versus national average)	2010	Increase in wealth index will increase redemption rates	TDHS

MEDA: Mennonite Economic Development Associates; NMCP: National Malaria Control Programme; THMIS: Tanzania HIV/AIDS Malaria Indicator Survey; TDHS: Tanzania Demography and Health Survey.

### Potential determinants of redemption rates/bed net use

Potential determinants of redemption rates/bed net use included: retailer-clinic ratio (ratio of the number of retailers to the number of clinics issuing vouchers in every region in a given year); top-up price (additional fee paid in cash by a woman redeeming a voucher for a LLIN at a retailer in each region in a given year); under-five coverage campaign (U5CC) (0/1: year in which free nets were distributed to every registered under-five child in every region between 2009 and 2010). Universal coverage campaign (0/1: year in which free nets were distributed to cover every registered sleeping space not covered by the under-five campaign between 2010 and 2011); prevalence of malaria (number of positive cases detected by microscopy (and/or rapid diagnostic test) divided by the total number of individuals in each regional sample); mass media as a form of behavioural change communication (BCC) activity of the National Malaria Control Programme (NMCP): percentage of men and women exposed to at least one source of mass media in one week, and relative wealth index (socio-economic score assigned to each region based on their wealth as compared to the national average). Data sources included MEDA, NMCP, Tanzania HIV Malaria Indicator Surveys (THMIS) [2], and Tanzania Demography and Health Surveys (TDHS) [21]. These potential determinants, data sources and years of availability are shown in Table 2, along with their presumed effect on redemption rates.

### Data collection

The redemption rates for both voucher types were computed from the MEDA database. The retailer-clinic ratios were also computed from same source. Since MEDA coordinated the distribution of free nets during the under-5 and universal coverage campaigns, they also provided us with the detailed schedule of both campaigns.

Household survey and retail audit data (from surveys carried out by the Ifakara Health Institute) were sourced from the database of the NMCP. The average top-up price paid in surveyed districts in the different years was computed from the retail audit data. Since the samples of these surveys were sample district-based, the top-up prices in the sampled districts, for the period before 2009 when a fixed top-up was introduced, were applied as the regional prices and one or two non-surveyed regions in each year were given the national average top-up price for that year. These average top-up prices were converted to their dollar equivalent based on the average exchange rates for the respective years [22]. Bed net use data for all household members and under-5 children were sourced from the THMIS done in 2007 [2]. The same source also provided the regional malaria prevalence data in 2007 [2].

The regional relative wealth indices (in quintiles) was sourced from the 2010 TDHS [21]. This regional wealth index was computed using information on household assets at regional level and then derived by principal component analysis [21]. Asset information involved household ownership of certain items as well as housing characteristics. Each asset was assigned a score and each household was then assigned a summary score for each owned asset. The sample was then divided into quintiles [21] and each region put into an average wealth quintile in comparison to all other regions.

Finally, the average exposure of men and women to various means of mass media in one week were sourced from the 2010 TDHS [21].

### Data analyses

The outcome variables were summarized by year and region and the effects of potential determinants on selected outcomes were analysed using a generalized estimating equation (GEE) model (binomial family for redemption rates and Gaussian family for bed net coverage). The models were selected to adjust for between-cluster variations. The voucher redemption rates, average top-up prices, retailer-clinic ratios and malaria prevalence rates were used as continuous quantitative variables. The campaigns were a NO (0) or YES (1). Prior to the campaign date, a ‘0’ was assigned to each region and a ‘1’ was assigned from the campaign year onward until the end of the study. For the net use model, the redemption rates were used as categorical variables.

The final model for the voucher redemption rate analysis included average top-up price, retailer-clinic ratio, UCC, U5CC, malaria prevalence, socio-economic status and exposure of men and women to mass media. The net use model additionally included the voucher redemption rates but did not include average top-up price and retailer-clinic ratio. All statistical analyses were done with STATA version 12 [Stata Corporation, Texas].

## Results

**Figure 2:**
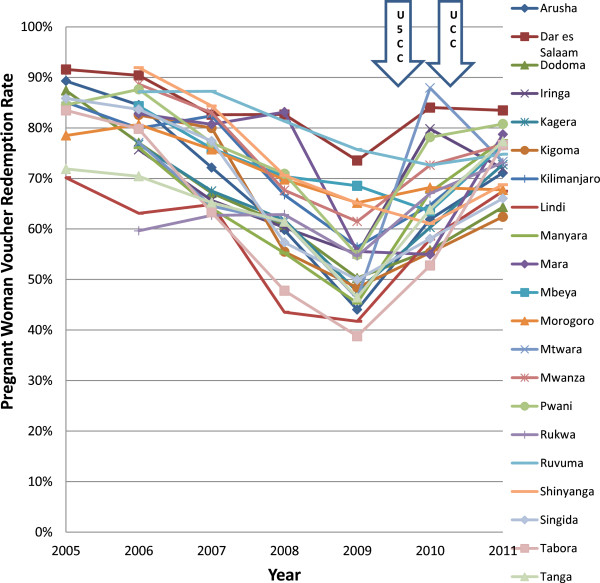
**Regional trends in pregnant woman voucher redemption rates.** PWV: Pregnant woman voucher; U5CC: Under-five coverage campaign; UCC: Universal coverage campaign.

**Figure 3:**
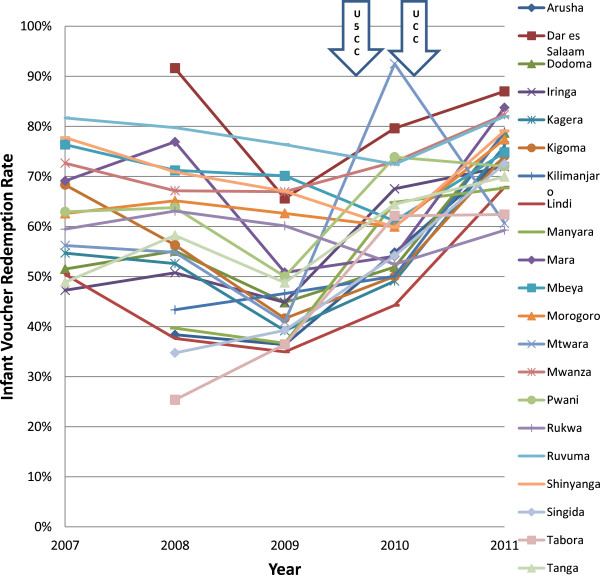
**Regional trends in infant voucher redemption rates.** IV: Infant voucher; U5CC: Under-five coverage campaign; UCC: Universal coverage campaign.

**Figure 4:**
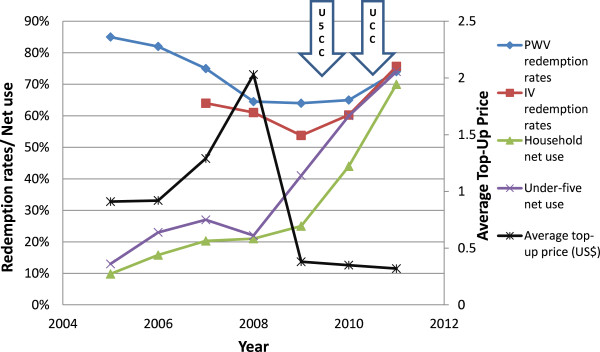
**Trends of study outcomes and interventions.** TOPUP- Average top-up price (USD); U5CC- Under-five coverage campaign; UCC- Universal coverage campaign; PWVRR- Pregnant woman voucher redemption rate; IVRR- Infant voucher redemption rate; HH- Household; U5- Under-five.

**Figure 5:**
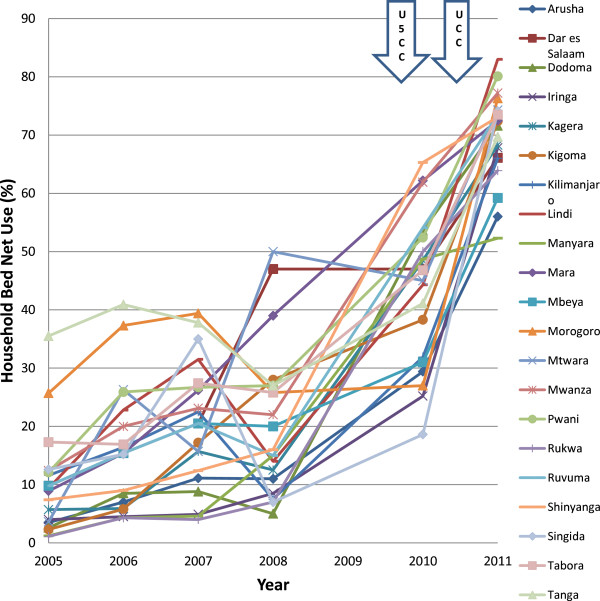
**Regional trends in household bed net use.** U5CC: Under-five coverage campaign; UCC: Universal coverage campaign.

**Figure 6:**
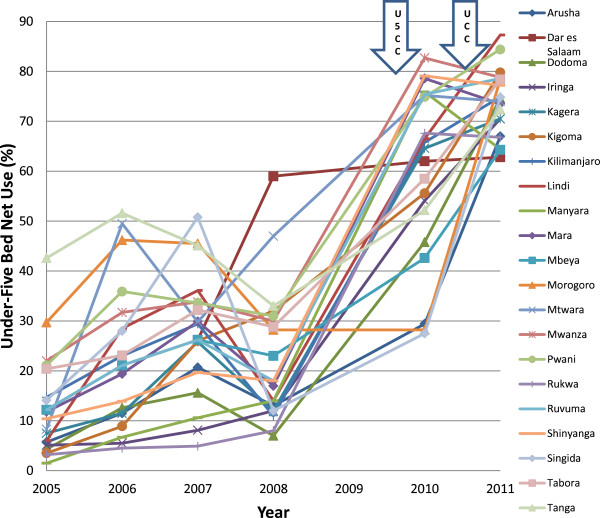
**Regional trends in under-five bed net use.** U5CC: Under-five coverage campaign; UCC: Universal coverage campaign.

The distribution of voucher redemption rates over the years of study is shown in Figures 2 and 3. There was a marked improvement in regional voucher redemption rates after 2009 (Figures 2 and 3). These improvements occurred following three major interventions: (1) a change towards a uniform low top-up of TSHS 500 (USD 0.40) in 2009, the U5CC in 2009 2010 and (3) the UCC in 2010 2011. Both the PWV and IV redemption rates increased up to >70% in 2011. This change corresponded to the reduction in top-up prices paid by the women after 2009 from about $1 to $0.40) (Figure 4) and this trend was seen regardless of the concurrent mass distributions of free nets. There were also marked improvements in the regional use data of bed nets, especially after 2009 (Figures 5 and 6). This coincided with both the change in top-up price for vouchers and both mass campaigns of free nets (Figure 4).

### Determinants of voucher redemption

The redemption of vouchers appeared to be influenced by the average top up price, retailer-clinic ratio and socio-economic status (Table 3). For every dollar increase in top up price, the pregnant woman voucher redemption rate decreased by 35%: OR = 0.65 (95% CI 0.45-0.94) whereas the infant voucher redemption rate decreased non-significantly by 6%: OR = 0.94 (95% CI 0.58-1.53). An increase in the retailer-clinic ratio by 1 increased the PWV redemption rate by 43%: OR = 1.43 (95% CI 1.31-1.57) whereas the IV redemption rate increased by 27%: OR = 1.27 (95% CI 1.17-1.40). An increase in one socioeconomic category increased IV redemption rate by 43%: OR = 1.43 (95% CI 1.10-1.70) and PWV redemption rate by 35%: OR = 1.35 (95% CI 1.02-1.73). Neither malaria prevalence nor exposure to mass media affected voucher redemption rates. Interestingly, the UCC seemed to independently improve redemption rates of the PWV: OR = 1.55 (95% CI 1.06-2.27) and also the IV redemption rates, although this was not statistically significant: OR = 1.26 (95% CI 0.88-1.80). Similar results were observed for the U5CC. For PWV redemption rates: OR = 1.18 (95% CI 0.92-1.54), hence a non-significant improvement, while it was significant for IV redemption rates: OR = 1.44 (95% CI 1.03-2.01).

**Table 3:** Determinants of voucher redemption rates

**Determinant**	**Outcome**	**Unadjusted OR (95% CI)**	**Fully adjusted OR (95% CI)**
Top-up price	PWV	1.15 (1.02-1.29)	0.65 (0.45-0.94)
	IV	0.73 (0.62-0.86)	0.94 (0.58-1.53)
Retailer-clinic ratio	PWV	1.01 (0.89-1.15)	1.43 (1.31-1.57)
	IV	0.82 (0.70-0.96)	1.27 (1.17-1.40)
Universal coverage campaign	PWV	1.11 (0.90-1.40)	1.55 (1.06-2.27)
	IV	2.06 (1.69-2.53)	1.26 (0.88-1.80)
Under-five coverage campaign	PWV	0.70 (0.60-0.82)	1.18 (0.92-1.54)
	IV	1.76 (1.42-2.20)	1.44 (1.03-2.01)
Malaria prevalence	PWV	1.01 (0.99-1.02)	1.01 (0.99-1.01)
	IV	0.99 (0.98-1.01)	1.01 (0.99-1.03)
Socio-economic status	PWV	1.33 (1.02-1.70)	1.35 (1.02-1.73)
	IV	1.39 (1.12-1.72)	1.43 (1.10-1.70)
Exposure of men to mass media	PWV	1.25 (0.92-1.70)	0.99 (0.98-1.01)
	IV	1.21 (0.87-1.69)	1.01 (0.99-1.01)
Exposure of women to mass media	PWV	1.19 (0.94-1.51)	1.01 (0.99-1.01)
	IV	1.17 (0.90-1.53)	1.00 (0.99-1.01)

PWV: pregnant woman voucher; IV: infant voucher; OR: odds ratio; CI: confidence interval. OR values represent per cent increase or decrease in voucher redemption rate, per unit increase in variable.

### Determinants of bed net use

The U5CC was a major determinant of bed net use, with OR = 1.71 (95% CI 1.07-4.88) for household net use and 3.28 (95% CI 2.09-5.16) for under-five bed net use (Table 4). Unexpectedly, this was not the case for the UCC. For households (all ages) OR = 1.16 (95% CI 0.77-1.72) and for under-fives OR = 1.19 (95% CI 0.86-1.67). The use of treated bed nets by both population groups slightly varied by malaria prevalence, increasing by 2% for every 1% increase in malaria prevalence but with only borderline significance: OR = 1.02 (95% CI 1.00-1.03). The socio-economic status and exposure to mass media were not significant determinants of bed net use in our model.

**Table 4:** Determinants of bed net use

**Determinant**	**Net use**	**Unadjusted OR (95% CI)**	**Fully adjusted OR (95% CI)**
PWV redemption rate	HH	0.92 (0.73-1.15)	1.07 (0.87-1.32)
	U5	0.94 (0.76-1.18)	1.06 (0.86-1.30)
IV redemption rate	HH	1.46 (1.23-1.72)	1.06 (0.86-1.30)
	U5	1.39 (1.19-1.61)	1.05 (0.87-1.28)
Universal coverage campaign	HH	4.09 (3.29-5.10)	1.16 (0.77-1.72)
	U5	3.49 (2.82-4.31)	1.19 (0.86-1.67)
Under-five coverage campaign	HH	4.28 (3.26-5.61)	1.71 (1.07-4.88)
	U5	3.84 (2.96-4.99)	3.28 (2.09-5.16)
Malaria prevalence	HH	0.99 (0.98-1.01)	1.02 (1.00-1.03)
	U5	0.99 (0.98-1.01)	1.02 (1.00-1.03)
Socio-economic status	HH	1.22 (0.93-1.60)	1.15 (0.94-1.41)
	U5	1.19 (0.93-1.53)	1.16 (0.99-1.01)
Exposure of men to mass media	HH	1.27 (0.16-1.69)	1.01 (0.98-1.05)
	U5	1.24 (1.00-1.03)	1.00 (0.99-1.01)
Exposure of women to mass media	HH	1.20 (0.98-1.47)	1.00 (0.99-1.01)
	U5	1.19 (0.99-1.42)	1.01 (0.98-1.05)

HH: Household   i.e. all ages; U5: Under-five; OR: odds ratio; CI: confidence interval. OR values represent per cent increase or decrease in voucher redemption rate, per unit increase in variable.

## Discussion

### Determinants of pregnant woman voucher redemption rate

The trend of the redemption rates over the years 2004 2011 we describe were in line with another evaluation done by the NMCP [23]. The consistent drop in redemption rates since 2005 was attributed to the rising top-up prices paid by women, up to an average of more than one dollar in 2009. When the top-up mechanism was transformed in the last quarter of 2009 into a fixed and much lower cost of TZS 500 (around USD 0.40 in 2009 and USD 0.30 in 2013) rates climbed steadily.

The retailer-clinic ratio (the average number of participant net retailer per voucher issuing clinic) was also a significant determinant of redemption rates. For instance, if a region had two retailers for every clinic, the chances of voucher redemption would increase by 43% compared to a region having only one retailer on average. This confirmed the importance of a vibrant commercial sector for the successful running of the TNVS. The logistic contractor for the TNVS (MEDA) has a target of two registered retailers for every registered clinic participating in the TNVS and is working towards that goal. From a trend analysis as well as from the regression analysis, the top-up price was the most important factor in redemption rate variations over the years, regardless of national campaigns of free nets. The odds of voucher redemption were reduced by 35% for every $1 increase in top-up fee. This was consistent with findings from the 2006 qualitative survey done in Tanzania to assess reasons for non-redemption of vouchers by pregnant women, which showed inability to afford a high top-up as the major reason for non-redemption (53.2%). Other reasons included lack of voucher nets in shops (11%), loss of vouchers before redemption (10%), already had a net / don’t need a net (8%) and no knowledge of any retail shops (4.6%) [15].

Contrary to present hypotheses, and also contrary to the fears by NATNETS stakeholders, neither the U5CC nor the UCC had a detrimental impact on the voucher redemption rates. This is a very important finding, which shows that mass campaigns of free nets and routine distribution systems relying on some cost participation are compatible, a point hotly debated in the mid 2000’s [24,25]. One anecdotal reason for this might be that the small size and quality of the nets distributed during the campaigns were less popular among the population than the TNVS nets.

The absence of effect of the campaigns is also supported by the work of Jean-Richard [26], which showed that free nets distributed in Lindi and Mtwara regions in 2007 did not affect net sales in the commercial sector during that period. On the other side, a study by Gingrich *et al*. [27] to assess price and income elasticity of ITN demand suggested that free ITNs reduced demand for ITN by voucher recipients in the short term. This difference is best explained by the different data sources, covering different time periods.

### Strengths and limitations of the study

A major strength of the present study is that the available data volume was very was large and covering a wide time range. It is believed that the findings are robust as well as highly relevant, since they result from a comprehensive national sample. However, there were also several inconsistencies and obvious mistakes in the available data, such as a region having a PWV redemption rate of >1000% in a particular year. This is a drawback for reliance on routine data.

The regional clinic history did not go further back than 2010 in the MEDA quarterly reports. Hence, this history was worked out assuming no change in the clinic count, from the number that was registered at the start of the scheme. Also, the retailer count was sometimes inconsistent, as MEDA initially reported figures from the net suppliers rather than their own counts. These numbers were noted to be inflated when MEDA began registration of retailers in 2010. Some regions had documented top-up prices even when the scheme had not been launched in those regions. Efforts were made to correct these errors in close collaboration with MEDA and the NMCP; there were no other ways of validating the remaining data. Clearly, data quality is important for better assessments and monitoring of the TNVS, and this needs to improve.

### Other problems of the TNVS

During its existence the TNVS was also facing operational problems, which affected the general performance of the scheme. These included funding gaps, voucher financial liability, insufficient clinics and retail outlets, LLIN stock-outs in retail shops and so on. Obviously, for a national-level programme over such a long time period this was to be expected. The retailers are still not numerous enough, partly because shop owners do not see the TZS 500 paid by the women/mothers as being enough as a profit [28]. This is especially a problem since nets are relatively expensive compared to other goods in a typical shop, and hence they represent a large capital outlay. As of March 2012, there were 5,604 registered retailers in the TNVS. But in the rural areas, the aim to have at least 90% of the villages with at least one retail shop to redeem vouchers has not yet been achieved, let alone having two. This usually generates extra costs for the voucher recipients to travel to participating retailers.

Not even all the clinics in Tanzania are registered with the TNVS. As of March 2012, there were 4,816 registered clinics, out of the estimated 7,000 health facilities in the country [29] The overall clinic-retailer ratio was 1.16 (target: 2 retailers per clinic).

A-Z Textiles Limited, producer of the Olyset net, has been the sole supplier of LLINs for the NATNETS programme since 2009. This has created a monopoly and removed competition from the supply of LLINs to registered retailers, limiting choice and possibly leading to higher prices. Some retailers in the rural areas complained about not seeing the suppliers for over one year, thus making them unable to stock LLINs.

Despite these problems, the TNVS has achieved a lot in ensuring access to inexpensive LLINs among the vulnerable groups. It provides an effective “keep-up” mechanism, with an outreach record matched by few other programmes in sub-Saharan Africa. Since Tanzania is the only endemic country known to operate such a national-level voucher scheme with a strong private sector component (and hence cost-sharing), the lessons for other programmes are limited. But the demonstration on this large scale of the great price elasticity of demand (beyond USD 1 the uptake of LLINs is greatly reduced) could be useful to any other programme aiming to develop a commercial sector approach.

The main finding of the present study that voucher redemption rates were not harmed by the two massive campaigns of free nets shows that the different NATNETS components are synergistic rather than antagonistic. This suggests that free campaigns and continuous distribution systems such as the voucher scheme can continue to co-exist in the future, but ongoing evaluation of the complementarity of different distribution mechanisms remains essential.

## Abbreviations

AIDS: Acquired immunodeficiency syndrome; BCC: Behavioural change communication; DMO: District medical officer; e-voucher: Electronic voucher; GEE: Generalized estimating equations; HH: Household; HIV: Human immunodeficiency virus; ITN: Insecticide-treated nets; IV: Infant voucher; LLIN: Long-lasting insecticidal nets; MEDA: Mennonite economic development associates; NATNETS: Tanzanian national nets programme; NMCP: National malaria control programme; OR: Odds ratio; PWV: Pregnant woman voucher; SDC: Swiss agency for development and cooperation; SMITN: Social marketing of insecticide-treated nets; TDHS: Tanzania Demography and health survey; THMIS: Tanzania HIV/AIDS malaria indicator survey; TNVS: Tanzanian national voucher scheme; U5CC: Under-5 coverage campaign; UCC: Universal coverage campaign.

## Competing interests

The authors hereby declare no competing financial or non-financial interests.

## Authors’ contributions

The authors contributed equally to the conception of this study and the development of this manuscript. All authors read and approved the final manuscript.
